# Genome-wide identification, phylogeny, and expression analysis of PEBP gene family in *Castanea mollissima*

**DOI:** 10.3389/fgene.2025.1530910

**Published:** 2025-03-26

**Authors:** Yujuan Tian, Jinxin Wang, Xiangyu Wang, Dongsheng Wang, Xuan Wang, Jing Liu, Haie Zhang, Jingzheng Zhang, Liyang Yu

**Affiliations:** ^1^ Engineering Research Center of Chestnut Industry Technology, Ministry of Education, Hebei Normal University of Science and Technology, Qinhuangdao, Hebei, China; ^2^ Shijiazhuang Institute of Pomology, Hebei Academy of Agriculture and Forestry Sciences, Shijiazhuang, Hebei, China; ^3^ The Office of Scientific Research, Hebei Normal University of Science and Technology, Qinhuangdao, Hebei, China

**Keywords:** PEBP gene family, *Castanea mollissima*, phylogeny, expression analysis, RT-qPCR

## Abstract

The phosphatidylethanolamine binding protein (PEBP) family plays an important part in growth and development of plants. *Castanea mollissima* is an economic plant with significant financial value and has become an important food source in the Northern Hemisphere. However, the *PEBP* genes in *C. mollissima* have not been studied yet. In this study, six *PEBP* genes (*CmPEBP1* ∼ *CmPEBP6*) were identified in *C. mollissima* and comprehensively analyzed in terms of physicochemical properties, phylogeny, gene structures, *cis*-regulatory elements (*CREs*), transcription factor interaction, and expression profiles. The six *CmPEBP* genes were categorized into three subfamilies according to the phylogeny analysis, and all of them share extremely similar gene and protein structures. A total of 136 *CREs* were identified in the promoter regions of the *CmPEBP* genes, mainly related to growth and development, environmental stress, hormone response, and light response. Comparative genomic analysis indicated that the expansion of the *CmPEBP* genes was mainly driven by dispersed duplication, and the *CmPEBP3*/*CmPEBP5* derived from eudicot common hexaploidization (ECH) events retained orthologous genes in all species studied. A total of 259 transcription factors (TFs) belonging to 39 families were predicted to be regulators of *CmPEBP* genes, and *CmPEBP4* was predicted to interact with the most TFs. The RNA-seq data analysis indicated the potential roles of *CmPEBP* genes in the ovule, bud, and flower development of *C. mollissima*, as well as in the response to temperature stress, drought stress, and the gall wasp *Dryocosmus kuriphilus* (GWDK) infestation. Additionally, the expression of *CmPEBP* genes in *C. mollissima* seed kernel development and their response to temperature stress were confirmed by RT-qPCR assays. This study gives references and directions for future in-depth studies of *PEBP* genes.

## Introduction

The phosphatidylethanolamine binding protein (PEBP) family is a highly conserved protein family that is widely presented in all three domains (Eukaryota, Bacteria, and Archaea) of the phylogenetic tree (Chautard et al., 2004; Dong et al., 2020). *PEBP* genes in plants were first discovered in *Arabidopsis thaliana* (Xu et al., 2022). By now, the presence of PEBP gene family has been extensively reported in a large number of plants, such as *Picea abies* (Liu Y. Y. et al., 2016), *Solanum lycopersicum* (Cao et al., 2015; Sun et al., 2023c), *Actinidia chinensis* (Varkonyi-Gasic et al., 2013), *Vitis vinifera* (Carmona et al., 2007), *Populus tremula* (Mohamed et al., 2010), *Zea mays* (Danilevskaya et al., 2008), *Oryza sativa* (Tamaki et al., 2007; Song et al., 2018). In general, *PEBP* genes can be divided into three subfamilies: FLOWERING LOCUS T-like (FT-like), TFL1 TERMINAL FLOWER1-like (TFL1-like), and MOTHER OF FT AND TFL1-like (MFT-like) (Jin et al., 2021; Sun et al., 2023c). Moreover, recent studies on more species have found the existence of the PEBP-like subfamily in the PEBP family (Zhang et al., 2016; Dong et al., 2020).

Due to their diverse functions concerning the growth and development of plants, the *PEBP* genes in plants participate in a wide variety of biological processes, such as hormone signal transduction, flower bud differentiation, and reproductive development (Zheng et al., 2016; Huang et al., 2024; Wu et al., 2024). For instances, the overexpression of *OsMFT1* delays the heading date of *O. sativa* and leads to remarkably increased number of spikelets and branches per panicle, while its knockout mutant leads to an earlier heading period and decreased number of spikelets per panicle (Song et al., 2018); four FT-like genes *SlSP5G*, *SlSP5G2*, *SlSP5G3*, and *SlSP3D* in *S. lycopersicum* are involved in the photoperiod effect of tomato flowering (Cao et al., 2015). *ZCN8* interacts with *DLF1* to regulate *Z. mays* inflorescence development (Meng et al., 2011); the overexpression of the *CorfloTFL1* gene in *A. thaliana*, a *PEBP* gene in *Cornus florida* delays plant flowering (Liu X. et al., 2016); it has been reported that *TaPEBP1*, *TaPEBP3* and *TaPEBP5* play essential roles in response of *Triticum aestivum* to drought, cold stress and heat stress (Dong et al., 2020); *Aradu80YRY*, *AraduYY72S*, and *AraduEHZ9Y* in *Arachis duranensis*, along with *AraipVEP8T* in *Arachis ipaensis*, are potentially crucial regulators of flowering time (Jin et al., 2019); the overexpression of the *FtFT1* and *FtFT3* genes promotes flowering and yield in *Fagopyrum tataricum* (Nie et al., 2024).

*Castanea mollissima* is a plant of *Castanea* Mill in the family Fagaceae, known for its tasty nuts (Huang et al., 2023; Zhang et al., 2023). The nutritional value of *C. mollissima* has received widespread attention due to its abundance in many varieties of nutrients such as starch, protein, fat, vitamins, minerals (calcium, iron, zinc, potassium), and bioactive substances (Wang et al., 2022). The abundance of nutrients in *C. mollissima* nuts gives them many health benefits, including but not limited to enhancing anti-inflammatory and antioxidant effects, preventing heart disease and stress, lowering blood lipids and blood sugar, preventing cardiovascular diseases, and improving digestive function (Chang et al., 2020; Wang et al., 2022; Zhang et al., 2022). Additionally, the antioxidant substances contained in *C. mollissima* also have specific effects on anti-aging and cancer prevention (Zhang et al., 2014). Due to the good adaptability to the environment, developed root system, and tall tree body of *C. mollissima*, it can grow under relatively harsh conditions, empowering its excellent abilities in windbreak, sand stabilization, and soil and water conservation. However, there are few reports on the gene families in *C. mollissima* associated with its growth, development, and stress resistance, which undoubtedly limits our understanding of this miraculous plant.

The vital roles of the *PEBP* genes in growth and development, metabolic regulation, and response to stress factors of plants have been extensively demonstrated, but have not been systematically studied for *C. mollissima*. In this study, we systematically analyzed *PEBP* genes in the *C. mollissima* genome. The analysis covered the chromosome locations, phylogenetic relationships, gene structures, conserved motifs, *cis*-regulatory elements (*CREs*), collinearity, interacting transcription factors (TFs), and protein three-dimensional structures. Additionally, the expression levels of *CmPEBP* genes in different tissues of *C. mollissima* and under various environmental stresses were analyzed using RNA-seq data. The RT-qPCR results validated the differential expression of *CmPEBP* genes during *C. mollissima* seed development and temperature stress. The present study provides information for future in-depth characterization of the potential roles of *PEBP* genes in *C. mollissima* in its growth, development, and response to stress factors.

## Results

### Identification and physicochemical properties of *PEBP* genes in *C. mollissima*

According to the results of HMM and BlastP searches, six *PEBP* genes were identified in the *C. mollissima* genome, renamed as *CmPEBP1* - *CmPEBP6* by their relative positions on the chromosome. The information of these *CmPEBP* genes and the physicochemical properties of the proteins encoded by them can be found in Supplementary Table S1. The CmPEBPs contain amino acid residues of 172 (CmPEBP4 and CmPEBP5) - 189 (CmPEBP3), with a molecular weight within the range of 18.88 kDa (CmPEBP4) to 21.34 kDa (CmPEBP3). They have maximum and minimum aliphatic indexes of 88.90 (CmPEBP4) and 74.66 (CmPEBP3), respectively. The grand average of hydropathicity of all these CmPEBPs is negative, suggesting their hydrophilic nature. All the CmPEBPs have a theoretical isoelectric point (pI) greater than seven; therefore, they are considered as alkaline. In addition, their instability index ranges from 37.14 (CmPEBP1) to 52.00 (CmPEBP5). The subcellular localization prediction results of the CmPEBP proteins indicate that they are all cytoplasmic proteins. The prediction results of the secondary structure of the CmPEBP proteins showed that all of them mainly contain random coils, accounting for more than 58% of the protein’s amino acid composition, while extended strands and alpha helices have a lower proportion, accounting for less than 42% (Supplementary Figure S1; Supplementary Table S2). In addition, no beta-turn was found in the CmPEBPs. Since the three-dimensional structure of proteins is closely related to their functions (Yang, 2008; Bhattacharya et al., 2017), the three-dimensional structures of the CmPEBPs were constructed based on SWISS-MODEL and AlphaFold3, and the results indicated that all of them share a similar three-dimensional structure, suggesting that they may serve as structural foundations for similar functions (Supplementary Figure S1).

### Phylogenetic analysis and classification of the *CmPEBP* genes

**FIGURE 1:**
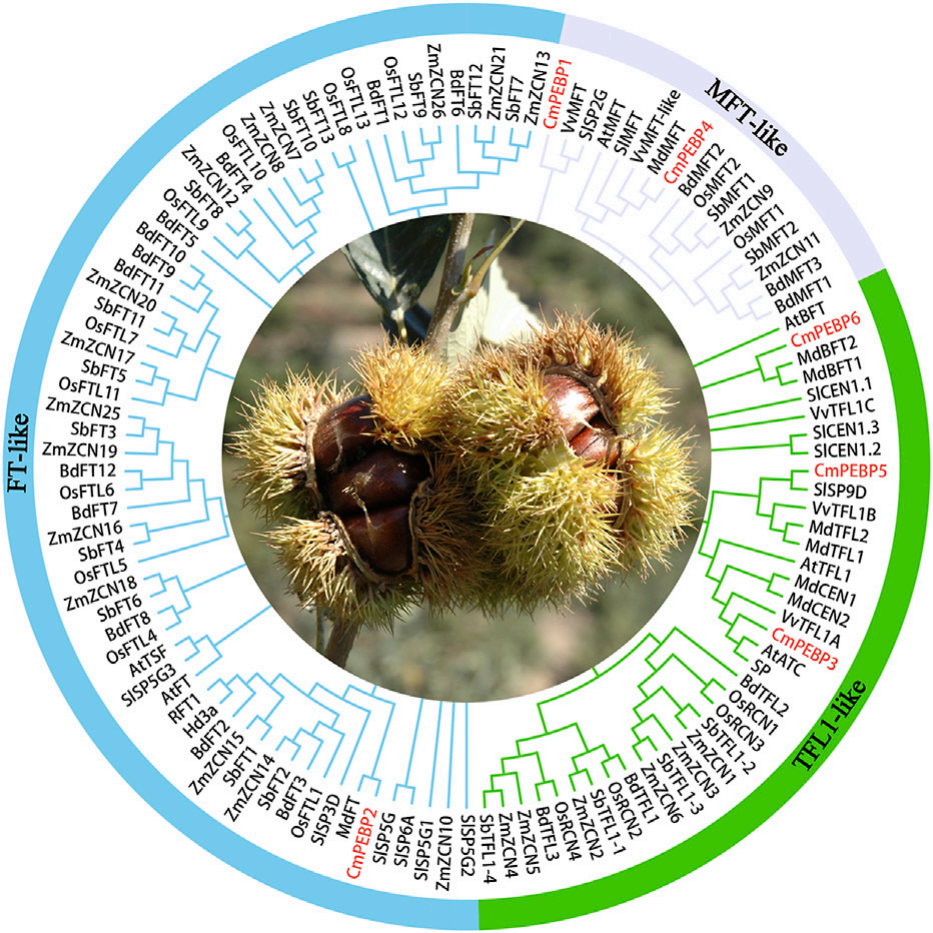
The phylogenetic tree of 117 PEBP proteins of *Arabidopsis thaliana* (6), *Malus domestica* (8), *Oryza sativa* (19), *Sorghum bicolor* (19), *Brachypodium distachyon* (18), *Solanum lycopersicum* (13), *Vitis vinifera* (5), *Zea mays* (23) and *C. mollissima* (6). MEGA 7.0 was used to construct the phylogenetic tree based on the protein sequences with the maximum likelihood method. The proteins were clustered into three groups.

To investigate the classification and evolution of the *CmPEBP* genes, the sequences of 117 PEBPs from *A. thaliana* (6), *Malus domestica* (8), *O*. *sativa* (19), *Sorghum bicolor* (19), *Brachypodium distachyon* (18), *S. lycopersicum* (13), *V. vinifera* (5), *Z. mays* (23), and *C. mollissima* (6) were used to construct a phylogenetic tree. As a result, the 117 PEBPs were classified into MFT-like, TFL1-like, and FT-like subfamilies (Figure 1). FT-like subfamilies have more PEBP members, and monocotyledonous plants tend to have more PEBP gene family members. For *C. mollissima*, the six CmPEBPs show an approximately uniform distribution in the three subfamilies; specifically, three of TFL1-like (CmPEBP3, CmPEBP5, CmPEBP6), two of MFT-like (CmPEBP1 and CmPEBP4), and one of FT-like (CmPEBP2).

### Analysis of gene structure, conserved motif, and chromosomal location of the *CmPEBP* genes

**FIGURE 2:**
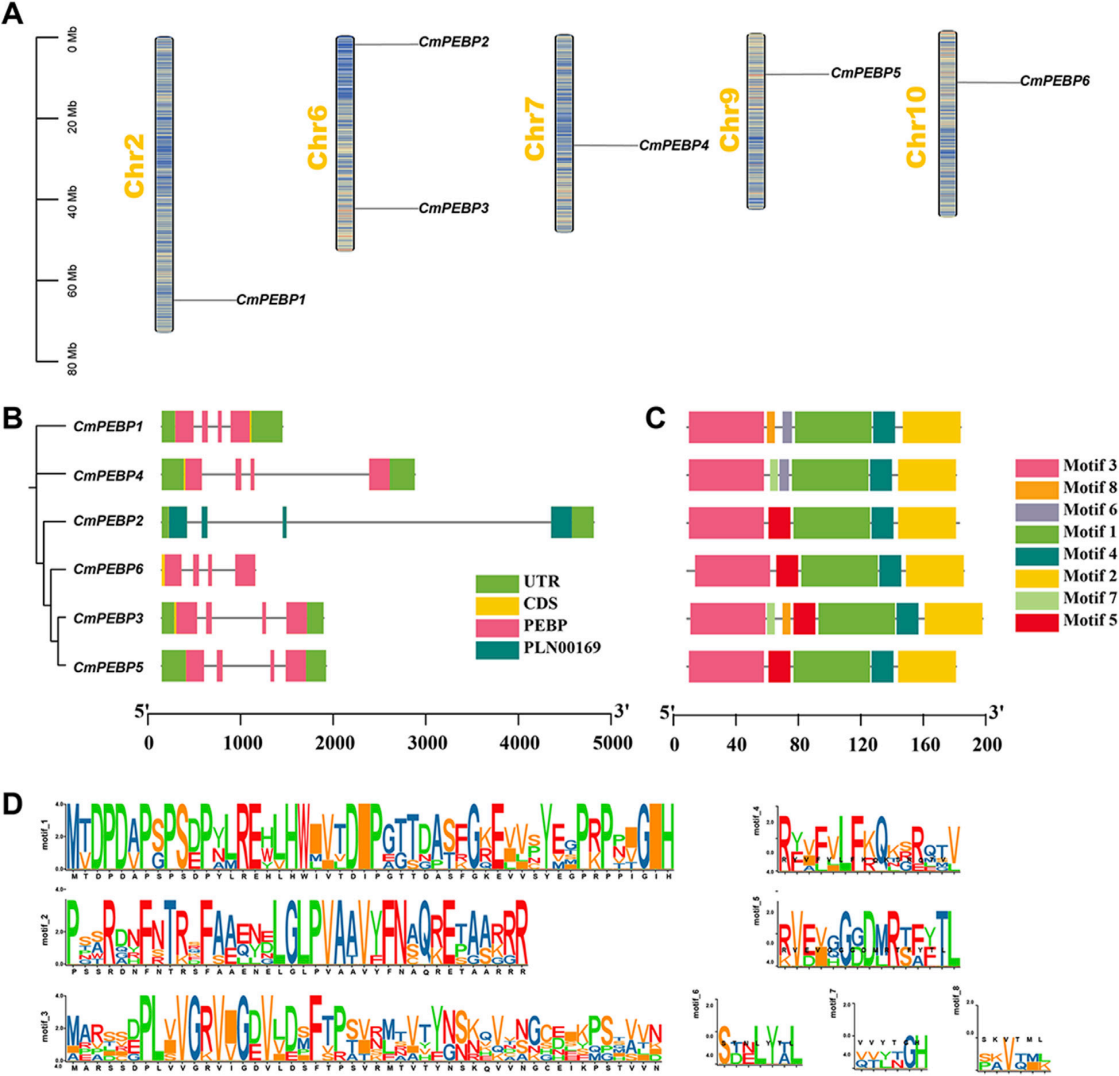
Chromosome distribution, gene structure, and conserved motifs of *CmPEBP* genes. **(A)** Chromosome distribution of *CmPEBP* genes. The color of segments in the chromosomes shows the gene density of the corresponding region. **(B)** Intron exon structure of *CmPEBP* genes. The phylogenetic tree containing only six *CmPEBP* genes is placed on the left side, constructed by MEGA 7.0 based on the maximum likelihood method. **(C)** Distribution of conserved motifs in CmPEBP proteins. **(D)** The sequence of eight conserved motifs in CmPEBP proteins.

The six *CmPEBP* genes are unevenly distributed on 5 *C. mollissima* chromosomes. Specifically, *CmPEBP2* and *CmPEBP3* are located on chromosome 6, and the other four *CmPEBP* genes are located on chromosomes 2, 7, 9, and 10, respectively (Figure 2A). Phylogenetic analysis classified the six *CmPEBP* genes into three subfamilies (Figure 2B). Furthermore, an analysis on the exon-intron structure of the *CmPEBP* genes was performed to investigate their gene structure. Interestingly, all the *CmPEBP* genes contain four exons and three introns for each, indicating the strong conservation gene structure of the *CmPEBP* genes (Figure 2B). Similarly, the conserved structural motifs of the proteins encoded by the *CmPEBP* genes were further investigated to understand the structural and functional characteristics of these genes (Figures 2C, D). As a result, eight conserved structural motifs (motif 1–8) were identified in the CmPEBPs, with five to seven conserved structural motifs in each CmPEBP. Specifically, motifs 1–4 are distributed in all the CmPEBPs and are arranged in the same order, suggesting that these four motifs are strongly conserved in the CmPEBPs; motif six is presented only in the MFT-like subfamily, while motifs 7–8 are distributed in both MFT-like and TFL1-like subfamilies. Overall, the CmPEBPs of the same subfamily tend to have the same conserved structural motifs.

### Analysis of *cis*-regulatory elements in promoter regions of the *CmPEBP* genes

**FIGURE 3:**
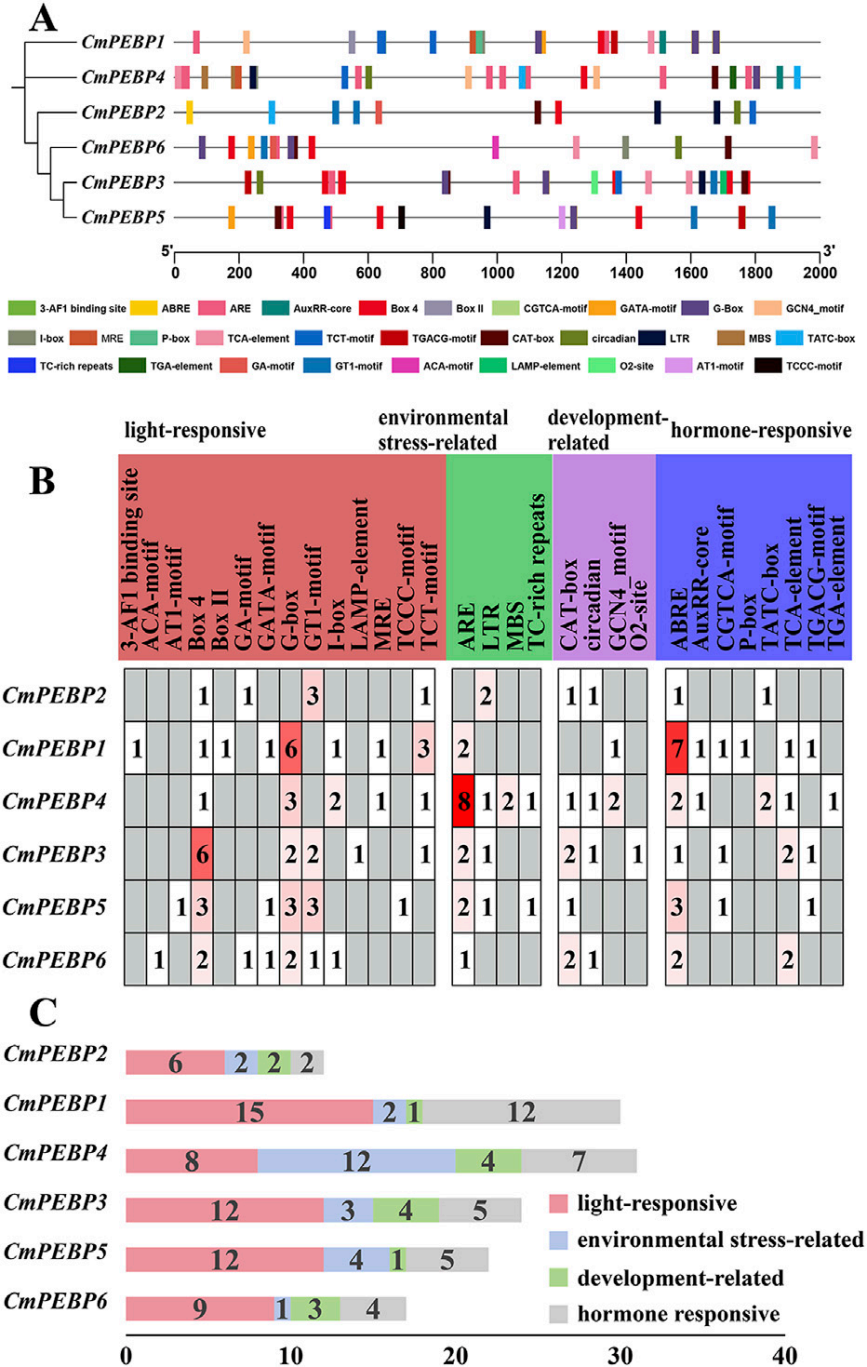
Prediction of *cis*-regulatory elements in the promoters of *PEBP* genes in *C. mollissima*. **(A)** *Cis*-regulatory elements in the promoters of six *CmPEBP* genes. Various color symbols present different elements, and their position in the figure indicates their relative position on the promoter. **(B)** The relative proportions of different *cis*-regulatory elements in the promoters of six *CmPEBP* genes are indicated in the chart. The same color represents *cis*-regulatory elements sharing identical or similar functions. **(C)** The number of various *cis*-regulatory elements in the promoters of each *CmPEBP* genes.

The analysis of *CREs* in a gene’s promoter regions helps to understand a gene’s potential functions (Wittkopp and Kalay, 2012). PlantCARE (https://bioinformatics.psb.ugent.be/webtools/plantcare/html/) was used to analyze the upstream DNA region of 2000 bp of the ATG (methionine) start codon of the *CmPEBP* genes to detect potential *CREs* (Figure 3A; Supplementary Table S3). As a result, 136 *CREs* in total were identified in the promoter regions of the six *CmPEBP* genes, which can be categorized into four types: 15 development-related *CREs*, 24 environmental stress-related *CREs*, 35 hormone-responsive *CREs*, and 62 photoresponsive *CREs* (Figures 3B, C). Notably, some CREs were distributed in almost all promoter regions of the *CmPEBP* genes (Figure 3). For example, Box four and G-box elements associated with light response were identified in the promoter region of six *CmPEBP* genes. ARE elements associated with environmental stress were identified in the promoter regions of five *CmPEBP* genes. CAT-box elements associated with growth and development were identified in the promoter regions of five *CmPEBP* genes. ABRE elements associated with hormonal responses were identified in the promoter regions of six *CmPEBP* genes.The analysis of promoter *CREs* suggested the vital roles of the PEBP gene family in hormone regulation, response to light signals, and resistance to abiotic stress.

### Collinearity analysis and duplication types

**FIGURE 4:**
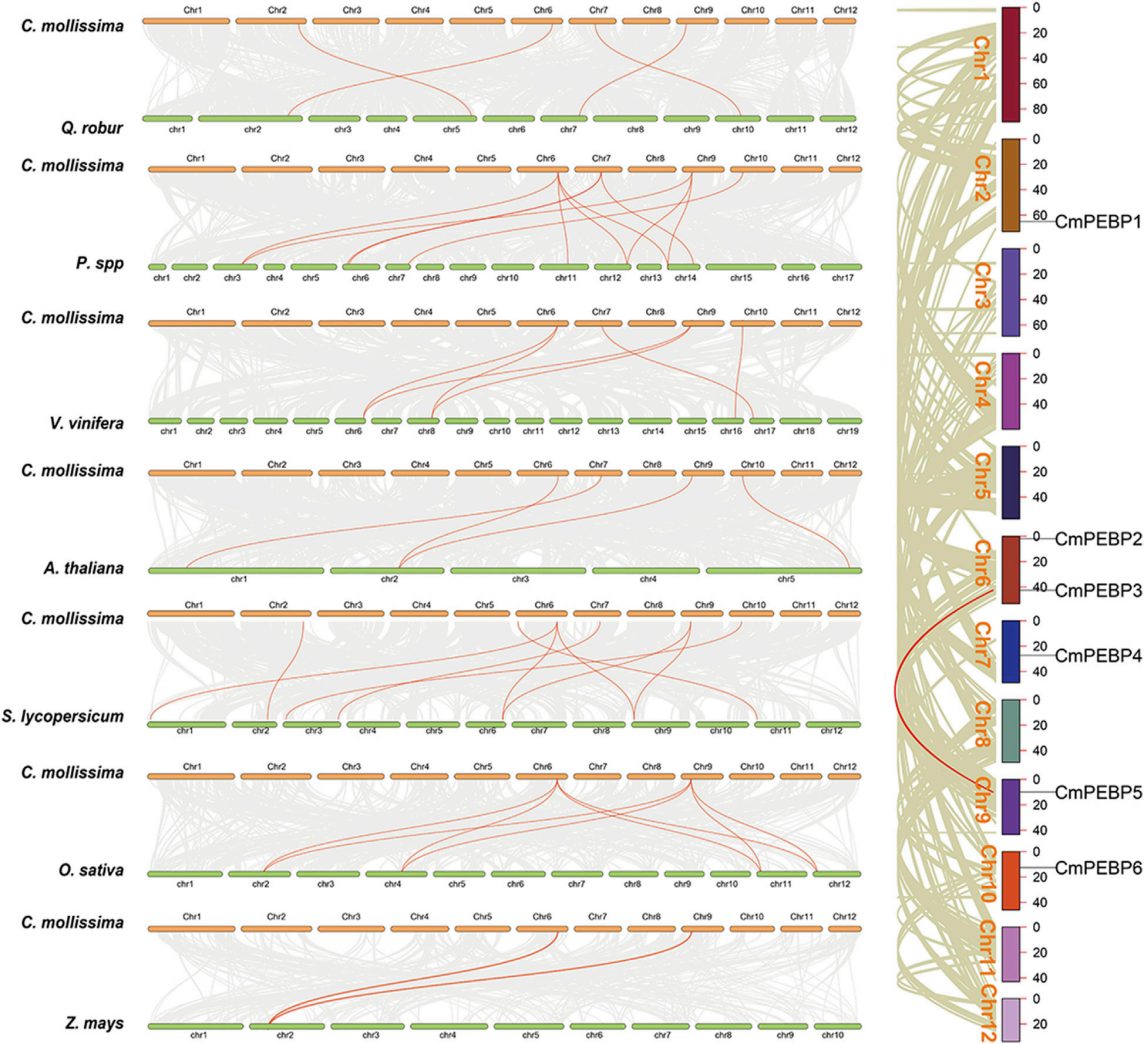
Collinearity analyses of *PEBP* genes within *C. mollissima* genome, and between the *PEBP* genes of *C. mollissima* and seven representative plant species (*Quercus*, *Pyrus*, *V. vinifera*, *A. thaliana*, *S. lycopersicum*, *O. sativa*, and *Z. mays*).

The collinearity between *C. mollissima* and seven representative species (five dicotyledonous plants: *A. thaliana*, *Quercus*, *V. vinifera*, *Pyrus*, and *S. lycopersicum*; and two monocotyledonous plants: *O. sativa* and *Z. mays*) was analyzed to understand the collinearity relationship of *PEBP* genes among different species (Figure 4). Four of the *CmPEBP* genes were identified in the collinear regions between *C. mollissima* and *V. vinifera*, *Pyrus*, *Quercus*, and *A. thaliana*, while all the six *CmPEBP* genes were identified in the collinear regions between *C. mollissima* and *S. lycopersicum* (Supplementary Tables S4–10). *CmPEBP3*, *CmPEBP4*, and *CmPEBP5* exist in the collinear region between *C. mollissima* and all five dicotyledonous plants. *CmPEBP3* and *CmPEBP5* were also found in the collinear regions of *C. mollissima* with *O. sativa* and *Zea may*. As indicated by these results, the orthologous genes of *CmPEBP3* and *CmPEBP5* are preserved in all these species, demonstrating their conservation in the evolution of the PEBP gene family. *CmPEBP3* and *CmPEBP5* both have three or more orthologous genes in *Pyrus* and *O. sativa*, suggesting their potentially essential roles in the evolution of the PEBP gene family based on the gene balance hypothesis (Birchler and Veitia, 2007). We further analyzed the length of collinear blocks containing the *CmPEBP* genes between *C. mollissima* and the seven representative species (Supplementary Tables S11–17). The median length of collinear blocks containing the *CmPEBP* genes was 48 (*C. mollissima* vs. *V. vinifera*), 51 (*C. mollissima* vs. *Pyrus*), 10.5 (*C. mollissima* vs. *Quercus*), 19 (*C. mollissima* vs. *A. thaliana*), 17.5 (*C. mollissima* vs. *O. sativa*), 16 (*C. mollissima* vs. *S. lycopersicum*) and 11.5 (*C. mollissima* vs. *Z. mays*) gene pairs, respectively. These data indicated that the *PEBP* genes are better preserved in the collinear regions of *C. mollissima*, *V. vinifera,* and *Pyrus* genomes, regardless of genome assembly quality.

**FIGURE 5:**
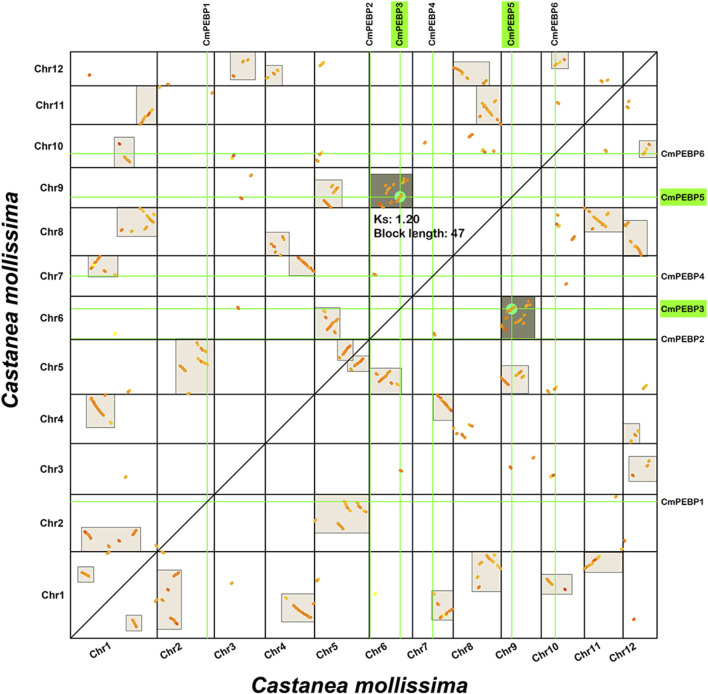
Homologous collinear dot-plot within the *C. mollissima* genome. The boxes in the figure represent collinear regions within the *C. mollissima* genome, in which the dark or light highlighted boxes indicate regions formed by WGD event containing *CmPEBP* homologous gene pairs and complementary fragments forming more significant homologous regions, respectively.

The important roles of gene duplication in gene family expansion and functional differentiation of genes have been widely reported (Liu et al., 2018; Quan et al., 2019). Therefore, the collinearity within *C. mollissima* genome was analyzed to explore the duplication type of the *CmPEBP* genes (Figure 4). The results indicated that *CmPEBP1*, *CmPEBP2*, *CmPEBP4*, and *CmPEBP6* originate from dispersed duplication, while *CmPEBP3* and *CmPEBP5* are believed to originate from whole genome duplication (WGD) or segmental duplication. *CmPEBP3* and *CmPEBP5* were identified to originate from the eudicot common hexaploidization (ECH) events through an analysis on the collinear homologous gene dot plot of *C. mollissima* genome, as previously demonstrated (Figure 5) (Yu et al., 2022a; Yu et al., 2023b).

### Interaction network of CmPEBP proteins and TFs regulatory network analysis

Seven proteins were predicted to interact with the CmPEBP proteins (Supplementary Figure S2). Specifically, two bZIP transcription factors interact with CmPEBP2, while three MADS-box proteins act on CmPEBP5; additionally, an unknown protein GWHTANWH026364 was predicted to interact with CmPEBP1, CmPEBP3, CmPEBP4, and CmPEBP6. The collinearity analysis showed that *CmPEBP5* retained three and four orthologous genes in the *Pyrus* and *O. sativa* genomes, respectively, significantly higher than other *CmPEBP* members (Supplementary Table S5; Supplementary Table S8). The gene balance hypothesis suggests that genes participating in macromolecular complexes or signaling networks are more likely to be preserved in evolution (Birchler and Veitia, 2007; Xu et al., 2016). Interestingly, *CmPEBP5* was predicted to interact with more other protein members, which is consistent with the interpretation of collinearity analysis results based on the gene balance hypothesis. In addition, numerous studies have demonstrated the interaction between bZIP transcription factors and FT-like proteins (Abe et al., 2005; Nan et al., 2014; Collani et al., 2019), MADS-box proteins act on TFL1-like proteins to regulate plant flowering (Liu et al., 2020). Interestingly, *CmPEBP2* and *CmPEBP5* belong to the FT-like and TFL1-like subfamilies, respectively (Figure 1), and have been predicted to interact with bZIP transcription factors and MADS-box proteins, respectively (Supplementary Figure S2A). These results further indicated the existence of interactions between CmPEBP proteins and the predicted proteins. Furthermore, the TFs that may interact upstream with the *CmPEBP* genes were predicted for further understanding of the potential functions of the *CmPEBP* genes (Figure 2B). As a result, 259 TFs were predicted to potentially act on the promoter regions of the *CmPEBP* genes, belonging to 39 TF families, such as MYB, NAC, SRS, SBP, and ERF (Supplementary Table S18). Among these predicted TF families, ERF has the most members (38), followed by MYB (25), WRKY (23), NAC (21), and bHLH (18); and the least abundant member in the TFs gene family is only one, such as FAR1, CAMTA, GRAS, ARR-B, SBP. Among the *CmPEBP* genes, *CmPEBP4* interacts with 156 of the TFs, followed by *CmPEBP1* (152), *CmPEBP5* (97), *CmPEBP2* (86), *CmPEBP6* (81), and *CmPEBP3* (69). MYB, WRKY, NAC, and bHLH are significantly enriched in the TFs regulatory network of the CmPEBP gene family, indicating their potential vital roles in regulating the driving biological functions of the *CmPEBP* genes. These findings provide useful information for understanding the genes that interact with the *CmPEBP* genes.

### Differences in expression of the *CmPEBP* genes

**FIGURE 6:**
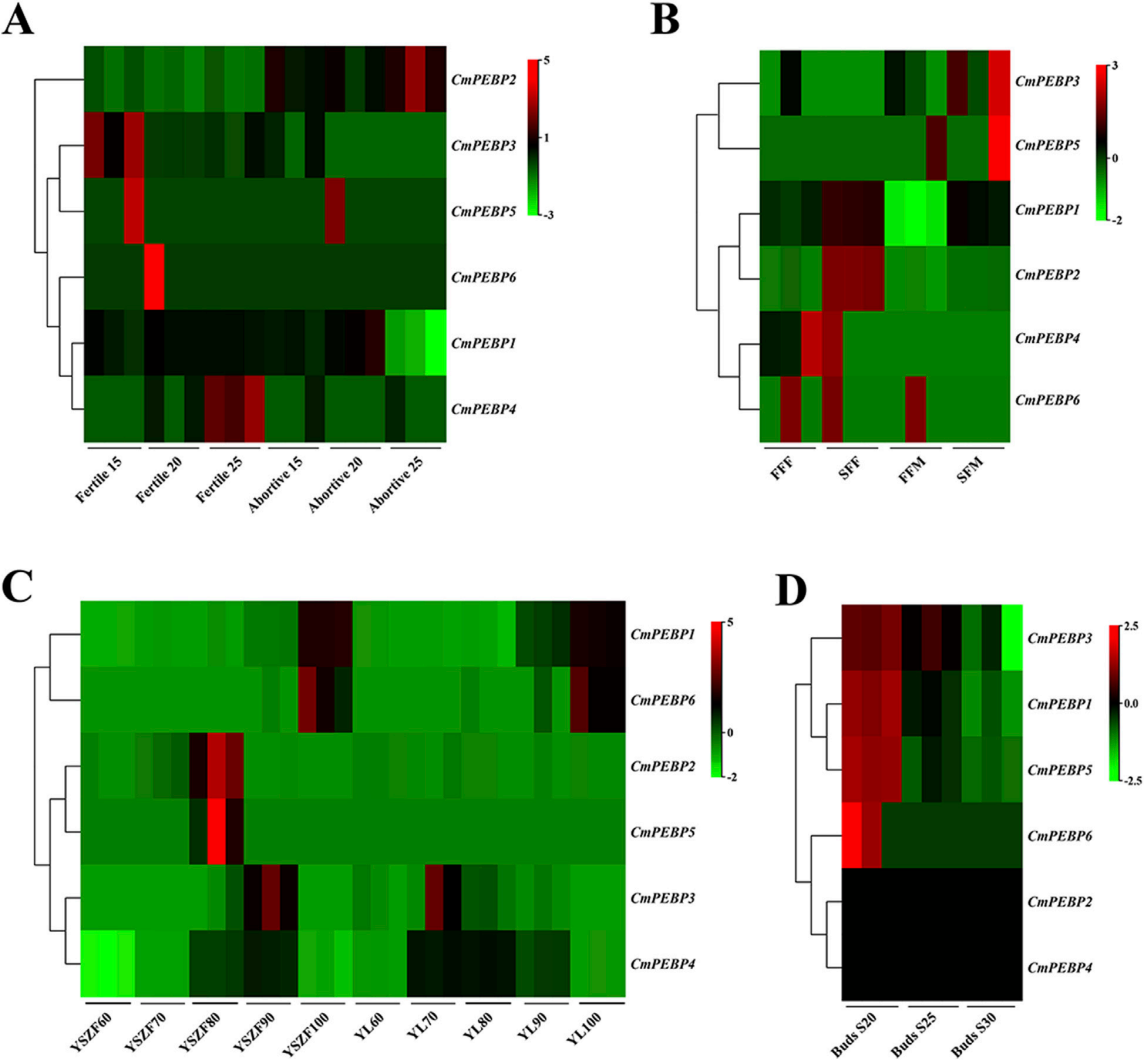
Genes expression of *CmPEBP* genes in different tissues of *C. mollissima*. **(A)** Genes expression in fertile and abortive ovules on 15-July, 20-July and 25-July. **(B)** Genes expression in first and second female flowering, first and second male flowering. FFF: First flowering (female), SFF: Secondary flowering (female), FFM: First flowering (male), SFM: Secondary flowering (male). **(C)** Genes expression in nuts of the cultivar “Yanshanzaofeng” and “Yanlong” 60, 70, 80, 90, and 100 days after flowering. **(D)** Genes expression in buds 20, 25, and 30 days after flowering.

To explore the potential functions of *CmPEBP* genes, the RNA-seq data of *C. mollissima* from NCBI was analyzed, including ovules (three periods of development for fertile and abortive ovules), flowers (primary and secondary, male and female flowers), seed kernels (five periods of development for two varieties), and buds (three periods of development) (Figure 6; Supplementary Tables S19–22). Five of the *CmPEBP* genes showed almost no expression at all three stages of development of ovules (fertile and aborted ovules) (FPKM < 2), with only *CmPEBP1* showing a significant decrease in expression levels during the late development of abortive ovules and in primary male flowers (Figures 6A, B; Supplementary Tables S19, 20). The expression level of *CmPEBP1* significantly increased during kernel maturation in both 2 *C. mollissima* varieties (‘Yanshanzaofeng’ and ‘Yanlong’); the expression of *CmPEBP4* was increased considerably (FPKM > 450) during the middle stage of seed kernel development (80 and 90 days after anthesis) and significantly decreased at the end of development (100 days after anthesis) (Figure 6C; Supplementary Table S21). These results indicated the potential roles of *CmPEBP1* and *CmPEBP4* in developing *C. mollissima* seed kernels. During the bud development *C. mollissima*, only *CmPEBP1* and *CmPEBP3* showed a significant decrease in expression levels, while others showed almost no expression (FPKM < 2) (Figure 6D; Supplementary Table S22). Overall, the *CmPEBP* genes exhibited different expression levels in various tissues of *C. mollissima* or at different stages of development within the same tissue, suggesting that the *CmPEBP* functions in other tissues of *C. mollissima*.

**FIGURE 7:**
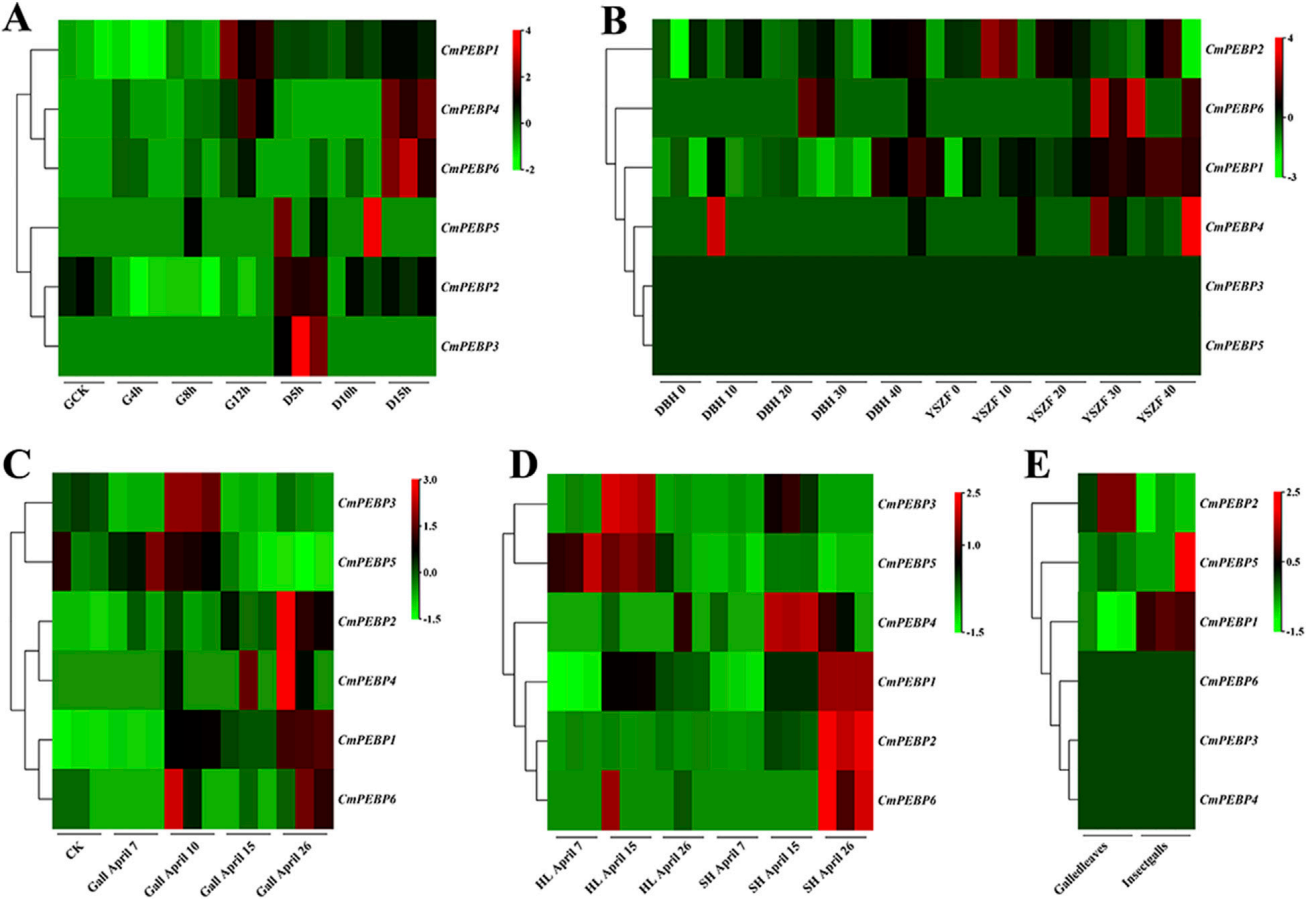
Genes expression of *CmPEBP* genes under different stresses in *C. mollissima*. **(A)** The expression profiles of CmPEBP genes under low (−15°C) and high (45°C) temperature stress at various periods. CK: control sample. G4h, G8h, G12h: high-temperature stress treatment for 4, 8, and 12 h, respectively. D5h, D10h, D15h: low-temperature stress treatment for 5, 10, and 15 h, respectively. **(B)** Genes expression in leaves of cultivar “Dabanhong” (DBH) and “Yanshanzaofeng” (YSZF) treated with drought for 0, 10, 20, 30, and 40 days. **(C)** Genes expression in leaves together with gall on 7-April, 10-April, 15-April, 26-April. **(D)** Genes expression in leaves with galls of cultivar “HongLi” (HL) (susceptible to GWDK infestation) and “Shuhe_Wuyingli” (SH) (partially resistant to GWDK infestation) infested with GWDK on 7-April, 15-April, 26-April. **(E)** Genes expression in leaves and insect galls induced by GWDK.

RNA-seq analysis was conducted on *C. mollissima* under the stress factors of low-temperature, high-temperature, drought, and GWDK infestation to understand the potential functions of the *CmPEBP* genes in coping with environmental stresses (Figure 7; Supplementary Tables S23–27). Under low-temperature stress, *CmPEBP1* showed a significantly increased expression at the beginning of the stress and always showed a high expression level throughout the stress, suggesting that it may be related to the resistance of *C. mollissima* resistance to low-temperature (Figure 7A; Supplementary Table S23). Under high-temperature stress, *CmPEBP1* showed a continuously increasing expression level and peaked at the end (Figure 7A; Supplementary Table S23). Under drought stress, *CmPEBP1* showed a significantly fluctuant expression, while the expression levels of all others of the *CmPEBP* genes showed almost no fluctuations, indicating that *CmPEBP1* may be involved in the resistance response of the plant to drought (Figure 7B; Supplementary Table S24). Additionally, the chestnut gall wasp, *Dryocosmus kuriphilus* (Hymenoptera Cynipidae), is a significant pest of cultivated *C. mollissima*, and therefore, the RNA-seq data of the *CmPEBP* genes in the galls formed by GWDK at different stages were analyzed. Compared with CK, the expression of *CmPEBP1* was remarkably upregulated in the galls of the initiation stage (7 April) formed by GWDK (Figure 7C; Supplementary Table S25). Furthermore, the expression level of *CmPEBP1* in *C. mollissima* variety ‘HongLi’ (susceptible to GWDK infestation) was significantly higher than that in variety ‘Shuhe-Wuyingli’ (partially resistant to GWDK infestation) in the galls (7 April) of the initiation stage formed by GWDK (Figure 7D; Supplementary Table S26); and notably, the expression level of *CmPEBP1* in galls formed by GWDK was significantly higher than that in *C. mollissima* leaves invaded by GWDK (Figure 7E; Supplementary Table S27). These results indicated the potential involvement of *CmPEBP1* in the development of galls formed by GWDK infestation. In summary, the CmPEBP gene family may be related to the response of *C. mollissima* to environmental stresses, but their specific functions in coping with environmental changes need further research.

### RT-qPCR of *CmPEBP* genes

**FIGURE 8:**
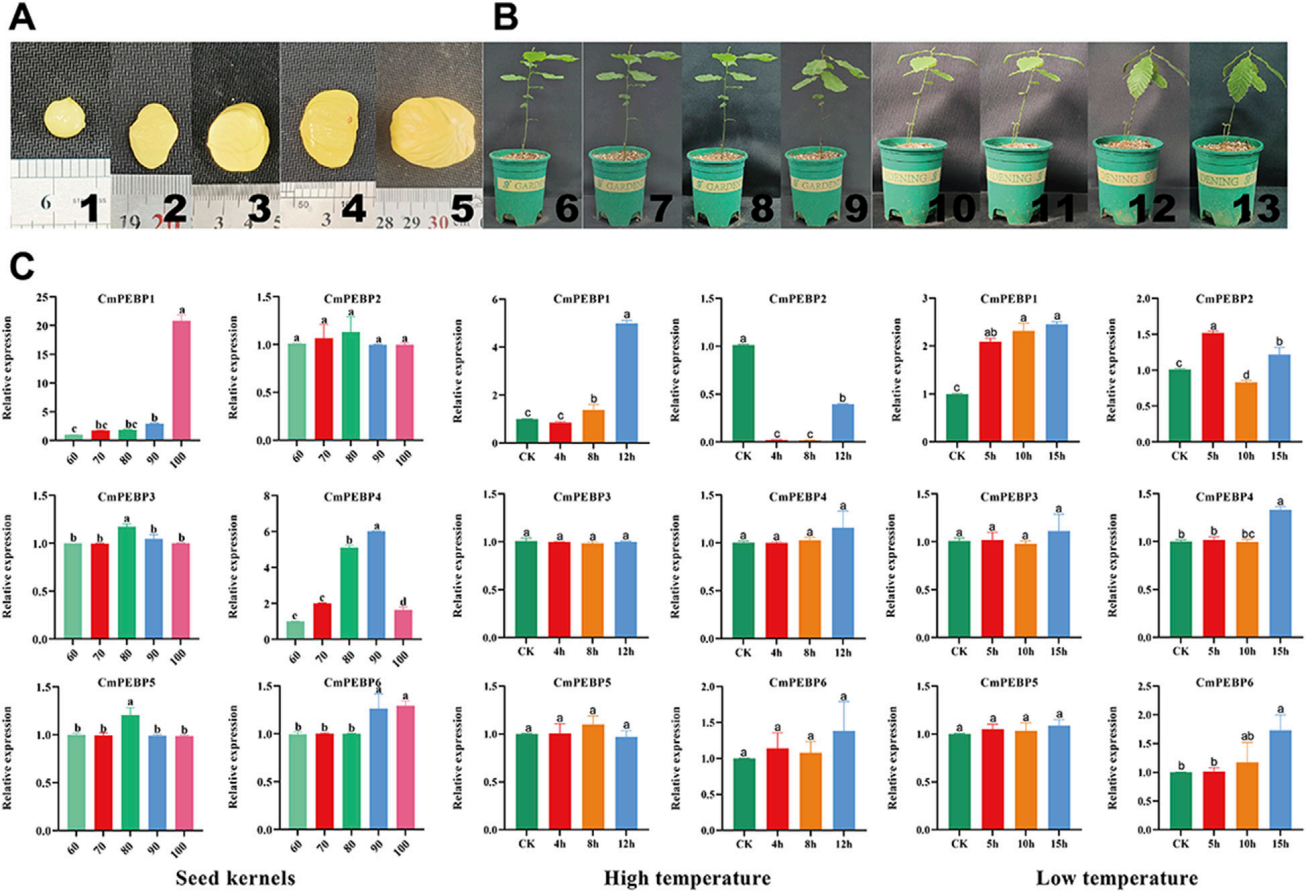
RT-qPCR of *CmPEBP* genes. **(A)** “Yanshanzaofeng” *C. mollissima* seed kernels, 1–5: seed kernels at 60, 70, 80, 90, and 100 days after flowering, respectively. **(B)** *C. mollissima* plants subjected to temperature stress treatment, 6∼9: *C. mollissima* plants were subjected to high-temperature stress for 0, 4, 8, and 12 h, respectively. 10∼13: *C. mollissima* plants were subjected to low-temperature stress for 0, 4, 8, and 12 h, respectively. **(C)** RT-qPCR of CmPEBP genes in *C. mollissima* leaves under high- and low-temperature stresses. Lowercase letter(s) above the bars indicate significant differences (α = 0.05, LSD) among the treatments.

For further verification of the confidence of RNA-seq data, RT-qPCR assays on the *CmPEBP* genes in leaves of *C. mollissima* plant at different stages of development of seed kernels and under varying degrees of temperature stress (Figure 8). The results showed that the expression level of *CmPEBP1* showed a trend of increasing with the development of *C. mollissima* seed kernels and peaked at 100 days after flowering. The expression level of *CmPEBP4* gradually increased during the early stage of development of *C. mollissima* seed kernels, peaked at 90 days after flowering, and was significantly downregulated in samples collected 100 days after flowering. The expression levels of all other four *CmPEBP* genes did not show significant changes during the development of *C. mollissima* seed kernels. In addition, *CmPEBP1* showed a significantly upregulated expression level under low-temperature stress, with a peak expression at D15h; *CmPEBP1* presented a significantly upregulated expression level under high-temperature stress, with a peak expression at G12h. There are strong correlation between RNA-seq and RT-qPCR results of six *CmPEBP* genes from kernel development, low-temperature stress, and high-temperature stress. Specifically, there is a significant positive correlation between the 12 group in the 18 sets of RNA-seq and RT-qPCR results (Supplementary Figure S3). Overall, RT-qPCR assays have demonstrated the expression of the *CmPEBP* genes in the development of *C. mollissima* seed kernels under temperature stress.

## Discussion

*C. mollissima* is an economically and ecologically important nut that plays important roles in food supply and ecosystem maintenance, especially in the Northern Hemisphere (Nie et al., 2021; Zhou et al., 2021; Hu et al., 2022). The diverse functions of the PEBP gene family in the growth and development of plants have been widely reported, such as regulating flowering time, controlling bud development and dormancy, and affecting plant light signal transduction (Danilevskaya et al., 2008; Liu Y. Y. et al., 2016; Yang et al., 2023). Conducting a systematic analysis of the PEBP gene family in *C. mollissima* PEBP can offer valuable information for disclosing the critical role of *PEBP* genes in the biological traits of *C. mollissima*. The PEBP gene family has been systematically characterized in a lot of plants, including *A. thaliana*, *O. sativa*, *Z. mays*, *S. lycopersicum*, *M. domestica.* (Chardon and Damerval, 2005; Danilevskaya et al., 2008; Karlgren et al., 2011; Yang et al., 2023). There have also been reports of the PEBP gene family in gymnosperms (Liu Y. Y. et al., 2016). Herein, six *PEBP* genes were identified in the *C. mollissima* genome, which can be classified into three subfamilies: MFT-like, TFL1-like, and FT-like (Figure 1). The number of members of the PEBP gene family is different from species, and this may be related to the duplication and retention of the PEBP gene family in species, which is ultimately reflected in related functions. All six identified *CmPEBP* genes contain four exons and three introns (Figure 2). Interestingly, all thirteen *PEBP* genes in *A. chinensis* and all five *PEBP* genes in *V. vinifera* also contain four exons and three introns (Carmona et al., 2007; Voogd et al., 2017). In addition, exon two and exon three of the *CmPEBP* genes identified in *C. mollissima* are shorter, while exon one and exon four are more extended, which is similar to the distribution of exon length of the *PEBP* genes found in some other species (Tsaftaris et al., 2013; Li et al., 2015). The three-dimensional structures of the six CmPEBPs are similar, and the two-dimensional structures are in similar proportions (Supplementary Figure S1). The collinearity analysis between *C. mollissima* and seven representative species showed that the orthologous genes of *CmPEBP3* and *CmPEBP5* were preserved in all eight species (Figure 4), suggesting the potential conservation of their role in the course of evolution.

The important roles of gene duplication in gene family expansion and functional differentiation have been widely reported (Panchy et al., 2016; De Smet et al., 2017; Pasquier et al., 2017). The wide variation in the number of members across species suggests that the PEBP gene family may have experienced complex gene duplication and gene loss during evolution (Liu Y. Y. et al., 2016; Sun et al., 2023c; Wu et al., 2024). The duplication patterns of the *CmPEBP* genes were investigated to enhance the understanding of the driving forces behind the expansion of this important gene family. The analysis results based on MCScanX indicated that four of six identified *CmPEBP* genes (*CmPEBP1*, *CmPEBP2*, *CmPEBP4*, and *CmPEBP6*) originated from dispersed duplication. Through duplication and dispersed insertion, dispersed duplication allows similar gene copies to expand in the genome, making it one of the main mechanisms for forming gene families (Innan and Kondrashov, 2010; Qiao et al., 2019; Chen et al., 2023). Members of gene families formed by dispersed duplication often undergo functional differentiation during evolution, which is also one of the ways to increase functional genomic diversity (Innan and Kondrashov, 2010; Qiao et al., 2019). Indeed, the four *CmPEBP* genes thought to originate from dispersed duplication showed tissue-specific expression and response to different stresses in transcriptome data analysis, suggesting their functional differentiation (Figure 6; Figure 7). In addition, *CmPEBP3* and *CmPEBP6* are thought to originate from WGD or segmental duplication. Since plant genomes are usually accompanied by fusion and recombination of chromosome segments after WGD, the types of WGD and segmental duplication are not distinguished (Yu et al., 2022a). Herein, *CmPEBP3* and *CmPEBP5* were identified as originating from WGD using a method that we have expertly used before, based on the complementarity of the collinear blocks formed by WGDs as well as their *Ks* values (Yu et al., 2022a; Yu et al., 2022b; Yu et al., 2023b) (Figure 6). The *C. mollissima* genome has not experienced additional WGD events after the ECH event (Sun et al., 2020; Sun W. et al., 2023), which also confirmed that *CmPEBP3* and *CmPEBP5* originate from the ECH event. In addition, in the collinearity analysis of *C. mollissima* genome with seven other representative species (five dicotyledonous and two monocotyledonous plants), it was found that the orthologous genes of *CmPEBP3* and *CmPEBP5* were preserved in the genomes of all the eight plant species, providing evidence that *CmPEBP3* and *CmPEBP5* have been well preserved in the evolution of angiosperms. Considering the above results, combined with the gene balance hypothesis, which suggests that genes involved in the formation of macromolecular complexes or signaling networks are more likely to be preserved in evolution (Birchler and Veitia, 2007), it is speculated that *CmPEBP3* and *CmPEBP5* play a vital part in the evolution of the PEBP gene family. The PEBP genes typically function through complex regulatory mechanisms to participate in plant growth, development, and response to environmental stresses (Karlgren et al., 2011; Zhao et al., 2020). Many *CREs* have been predicted in the promoter regions of the *CmPEBP* genes, including light-responsive, environmental stress-related, development-associated, and hormone-responsive elements (Figure 3). The abundance and quantity of light- and hormone-responsive elements indicate that the promoters of the *CmPEPB* genes are mostly inducible promoters, which can be regulated by light and hormone signals. In addition, *CREs* that respond to growth, development, and environmental stress have been predicted. For example, LTR (involved in response to low temperature), MBS (involved in inducibility in response to drought), and CAT-box (related to meristem expression). These results indicated the roles of the *CmPEBP* genes in the growth, development, and response to environmental stresses of *C. mollissima*. The RNA-seq data of the *CmPEBP* genes in multiple tissues of *C. mollissima* and under different stresses were analyzed to explore their potential functions (Figure 6; Figure 7). The expression level of *CmPEBP1* was significantly reduced during the late development of aborted ovules and in primary male flowers (Figures 6A, B). It was significantly upregulated during the maturation of *C. mollissima* seed kernels. In contrast, the expression level of *CmPEBP4* was significantly upregulated during the middle stage of development of seed kernels, suggesting that both *CmPEBP1* and *CmPEBP4* are involved in the maturation of *C. mollissima* seed kernels (Figure 6C). Moreover, the expression level of *CmPEBP1* was significantly decreased with the development of *C. mollissima* buds, suggesting that *CmPEBP1* may be associated with such development (Figure 6D). Both *CmPEBP1* and *CmPEBP4* are of the MFT-like subfamily (Figure 1). Current research has found that the main function of genes of the MFT-like subfamily is the regulation of flowering (Yoo et al., 2004; Yuan et al., 2022; Wu et al., 2024), regulation of germination of seed kernels (Danilevskaya et al., 2008; Karlgren et al., 2011), and control of flower bud differentiation (Zhao et al., 2020). For example, *ZCN8* and *DLF1* have been shown to interact with and regulate the development of *Z. mays* flowers (Meng et al., 2011); *AtMFT* can regulate the germination and normal growth of *A. thaliana* seeds by modulating ABA and GA signals (Yoo et al., 2004; Xi et al., 2010; Vaistij et al., 2013).

*CmPEBP1* showed a remarkably upregulated expression level in samples collected under low-temperature and drought stresses (Figure 7B), while the expression level of *CmPEBP1* was significantly upregulated under high-temperature stress (Figure 7A). This finding indicated the potential role of *CmPEBP1* in adapting *C. mollissima* to low-temperature and drought stresses. Furthermore, *CmPEBP1* is suggested to be related to the GWDK infestation on *C. mollissima*. For example, compared to CK, *CmPEBP1* showed an upregulated in the galls of the initiation stage (7 April) formed by GWDK (Figure 7C); the expression level of *CmPEBP1* in *C. mollissima* variety ‘HongLi’ (susceptible to GWDK infestation) was significantly higher than that in the variety ‘Shuhe-Wuyingli’ (partially resistant to GWDK infestation), in the galls (7 April) of the initiation stage formed by GWDK (Figure 7D). All these findings justify the inference that *CmPEBP1* may participate in the response of *C. mollissima* to GWDK infestation. In addition, temperature stress-related *CREs* (LTR) were identified in the promoter regions of *CmPEBP1* and *CmPEBP4* (Figure 3). Furthermore, the expression levels of *CmPEBP1* and *CmPEBP4* under both high and low-temperature stresses were verified by RT-qPCR assays, which were confirmed to be consistent with the results of RNA-seq data analysis (Figure 8). Considering the results of RNA-seq analysis and RT-qPCR assays of *CmPEBP1* under temperature stresses, as well as the fact that *CREs* are identified in their promoter regions, it is believed that it is an important candidate gene for the response of *C. mollissima* to temperature stresses.

Herein, six *PEBP* genes were identified in the *C. mollissima* genome and then comprehensively characterized in terms of physicochemical properties, chromosome distribution, phylogenetics, gene structure, conserved motifs, conserved domains, collinearity relationships, *CREs*, and TFs regulatory networks. The results indicate that the *CmPEBP* genes exhibited strong conservation in gene structure, conserved motifs, and protein structure. The expression profile of the *CmPEBP* genes indicate their potential roles in the development of ovules, buds, seed kernels, and flowers, as well as in response to low- and high-temperatures, and GWDK infestation of *C. mollissima*. The results of RT-qPCR assays confirmed the expression patterns at different stages of development of seed kernels and the response to temperature stresses of the *CmPEBP* genes. The study offers valuable information from a theoretical point of view for future in-depth research on the functions of the PEBP gene family in *C. mollissima*.

## Materials and methods

### Identification of *PEBP* genes in *C. mollissima*

The sequences of PEBPs in *A. thaliana* and the conserved domain of PEBP (PF01161) were sourced from the Arabidopsis Information Resource (https://www.arabidopsis.org/) and Pfam databases (https://www.ebi.ac.uk/interpro/entry/pfam/#table) (Xu et al., 2022), respectively. The genome data and annotation files of chestnut (N11-1) were downloaded from the Castanea Genome Database (http://castaneadb.net/) (Wang et al., 2020). Using the sequences of PEBPs in *A. thaliana* as the query sequences, the candidate genes in all protein sequences of *C. mollissima* were searched with BlastP (E-value ≤1.0 × e−5). The sequences of proteins in *C. mollissima* were searched using the HMMER3.0 software, and then the candidate genes were screened (Finn et al., 2011). All candidate sequences identified were submitted to Batch-CD to ensure the existence of PEBP conserved domains and ultimately confirm the *CmPEBP* genes (https://www.ncbi.nlm.nih.gov/Structure/bwrpsb/bwrpsb.cgi). The prediction of the physicochemical properties and subcellular localization of CmPEBPs were performed using ExPASy and Cell-PLoc, respectively (Chou and Shen, 2008), and the prediction of the secondary structure of CmPEBPs were performed using SOPMA (Geourjon and Deléage, 1995); and then the three-dimensional structures of the CmPEBPs were constructed using the Swiss model and AlphaFold 3 (Waterhouse et al., 2018; Abramson et al., 2024).

### Phylogenetic analysis and collinearity analysis

Based on publicly published articles, there are 111 *PEBP* genes have been identified in eight species, including *A. thaliana* (6), *M*. *domestica* (8), *O. sativa* (19), *S*. *bicolor* (19), *B*. *distachyon* (18), *S. lycopersicum* (13), *V. vinifera* (5), and *Z. mays* (23) (Yang et al., 2023). By combining the sequences of the above PEBPs with the sequences of six PEBPs from *C. mollissima* a total of 117 protein sequences were obtained. The ClustalW program was used to conduct multiple alignment of the full-length sequences of CmPEBP proteins. We used the “Find Best DNA/Protein Models (ML)” function in MEGA7.0 to obtain the best amino acid substitution model (partial deletion 95%). Finally, a phylogenetic tree was constructed with the maximum likelihood estimation and MEGA 7.0 software, with the follow parameters: Jones–Taylor–Thornton (JTT) model; Gamma Distributed (G); Partial deletion 95%; 1,000 bootstrap replications (Kumar et al., 2016). Using the genomes of seven representative species, including *Quercus*, *Pyrus*, *V. vinifera*, *A. thaliana*, *S. lycopersicum*, *O. sativa*, and *Z. mays*, downloaded from Phytozome database, and then the collinearity relationships between *C. mollissima* and these representative species were analyzed with MCScanX (Wang et al., 2012). “File Merge For MCScanX” function in TBtools was used to transfer the origin gff3 file to a format suitable for MCScanX operation that only contains chromosome, gene ID, gene start and end position information. The “duplicate_gene_classifier” in MCScanX software was used to obtain the duplication type of *CmPEBP* genes with default parameters, such as WGD or segmental, proximal, tandem, and dispersed. Furthermore, *CmPEBP* members formed by WGD events were identified, as we did before (Yu et al., 2023a; Cao et al., 2024). Specifically, the homologous collinear gene dot-plot within the *C. mollissima* genome was generated using TBtools. The non-synonymous (*Ka*) and synonymous substitution sites (*Ks*) values of homologous gene pairs was generated using the “add_ka_and_ks_to collinearity” function in MCScanX software. The median *Ks* values of collinear blocks were calculated by writing the script (Yu et al., 2022b). The collinear blocks in the homologous gene dot-plot were colored differently based on different median *Ks* values. Combined with the distribution of *Ks* corresponding to the WGD event that occurred in the *C. mollissima* genome before (Yu et al., 2022b), the complementarity of the collinear blocks, the *CmPEBP* genes formed by the WGD event were identified.

### Gene structure, conserved motif, and *CREs* analysis

The gene structure information of *CmPEBP* genes was obtained based on the GFF3 file of *C. mollissima*, and the CmPEBPs were submitted to the online tool MEME for conservative motif prediction (Bailey et al., 2009). For each of the *CmPEBP* genes, the upstream sequence of 2000 bp was extracted as the promoter region by TBtools, which was then submitted to PlantCARE to predict *CREs* in the promoter region. Then, the gene structure, conserved motifs, and *CREs* were visualized with TBtools (Chen et al., 2020).

### Transcription factors regulatory and protein-protein interaction network analysis

The Plant Transcriptional Regulatory Map (https://plantregmap.gao-lab.org/) was used to predict the TFs that may act on the 2000 bp upstream regions of *CmPEBP* genes (P-value ≤1e^−6^) (Hu X. et al., 2023; Yu et al., 2025). Since the *C. mollissima* genomic information is not yet available in the STRING (https://cn.string-db.org/) database, we conducted a protein-protein interaction network analysis to explore proteins that interact with CmPEBP proteins based on the homologs of the *CmPEBP* genes in *A*. *thaliana*. These homologous proteins were subjected to the STRING to obtain the interacting proteins with default parameters (Szklarczyk et al., 2019). Then, these protein sequences were aligned to obtain the homologous protein sequences in *C. mollissima*. This method of obtaining protein interaction networks has been widely reported and used (Chen et al., 2021; Yu et al., 2021; Hu M. et al., 2023; Sun et al., 2023b). TFs and protein interaction analysis results were visualized using the Cytoscape 3.9.1 (Kohl et al., 2011).

### Expression analysis of the *CmPEBP* genes

Transcriptomic data of *C. mollissima* in different tissues (ovules, flowers, seed kernels) and under various stress factors (high temperature, low temperature, drought, and GWDK infestation) were obtained from the NCBI database (Supplementary Table S28). Sratolkit 3.0 and Tophat2 software were used to align the reads to the reference genome (Chinese chestnut (N11-1) downloaded from Castanea Genome Database) and confirm the expression levels of *CmPEBP* genes, respectively (Goldberg et al., 2009; Kim et al., 2013). TBtools was used to standardize FPKM values (“Normalize” function in TBtools) and generate heatmaps based on log_2_ (FPKM+1) value conversion for comparison purposes.

### Plant materials and RT-qPCR validation

The seed kernels of *C. mollissima* cv.Yanshanzaofeng at different stages of development were collected for RT-qPCR assays in 2024. Specifically, the fruits of ‘Yanshanzaofeng’ were collected at 60, 70, 80, 90, and 100 days after flowering, and after removing spines and shells, the seed kernels were rapidly frozen with liquid nitrogen and finally stored in the freezer at −80°C before RT-qPCR assay of the *CmPEBP* genes. In addition, we planted the seeds of *C. mollissima* cv. Yanshanzaofeng in April 2024 and subjected the plants to temperature stress treatment 60 days after sowing. Specifically, 9 *C. mollissima* trees were subjected to high-temperature treatment at 45°C, and leaf samples were collected after 4, 8, and 12 h. Similarly, 9 *C. mollissima* trees were subjected to low-temperature treatment at −15°C, and leaf samples were collected after 5, 10, and 15 h, respectively. Three *C. mollissima* trees grown at 25°C were used as the control. After collection, all samples were rapidly frozen with liquid nitrogen and stored at −80°C before further use. Before RT-qPCR assays, RNA extraction and reverse transcription of RNA into single-stranded cDNA were performed using the RNAprep pure Plant Kit (Tiengen, Beijing, China) and the PrimeScript RT Master Mix (Takara Biotechnology Co., Beijing, China), respectively. The RT-qPCR assays were conducted on the ABI 7500 Real-Time PCR system (Applied Biosystems Inc., Foster City, CA, USA) using TB Green Premix Ex Taq (Takara). The instrument settings were: 95°C for 300 s; 40 PCR cycles, with each cycle set at 95°C for 10 s and 60°C for 30 s. The specific primer information was shown in Supplementary Table S29, in which the *18S* gene of *C. mollissima* was used as the reference gene. The RT-PCR primer efficiency was determined using the standard curve method (Svec et al., 2015; Zhang et al., 2021). Briefly, serial dilutions of template were used to generate a standard curve, and primer efficiency was calculated based on the resulting cycle threshold (CT) values. The calculation of relative gene expression values was completed using the comparative 2^-△△CT^ method and three biological replicates were performed.

## Data Availability

The data presented in the study can be found in the NCBI Sequence Read Archive (SRA) repository. The accession number(s) can be found in the article/Supplementary Material.

## Supplementary material

SUPPLEMENTARY FIGURE S1 The predicted three-dimensional structure (based on SWISS-MODEL and AlphaFold3) and secondary structure analysis of CmPEBP proteins.

SUPPLEMENTARY FIGURE S2 CmPEBP protein interaction network and TFs regulatory network analysis. **(A)** CmPEBP protein interaction network analysis. **(B)** TFs regulatory network analysis of *CmPEBP* genes.

SUPPLEMENTARY FIGURE S3 The correlation between RNA-seq and RT-qPCR results of six *CmPEBP* genes from kernel development, low-temperature stress, and high-temperature stress.
