# Supplementary figures and images for: Genome-wide identification, phylogeny, and expression analysis of PEBP gene family in Castanea mollissima
Source: Front Genet. 2025 Mar 26;16:1530910. doi: 10.3389/fgene.2025.1530910 (PMC11979240; doi:10.3389/fgene.2025.1530910)

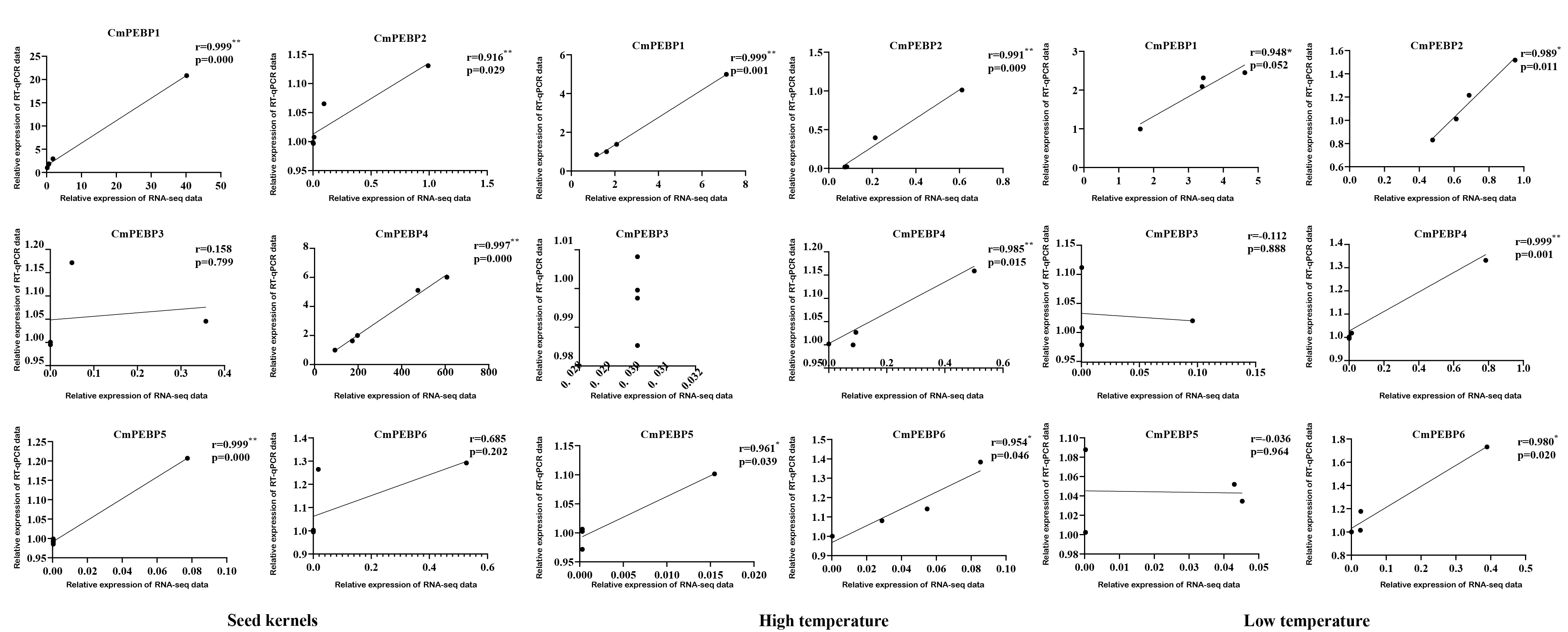

Supplement: Supplementary file 1 [file Image3.tif]

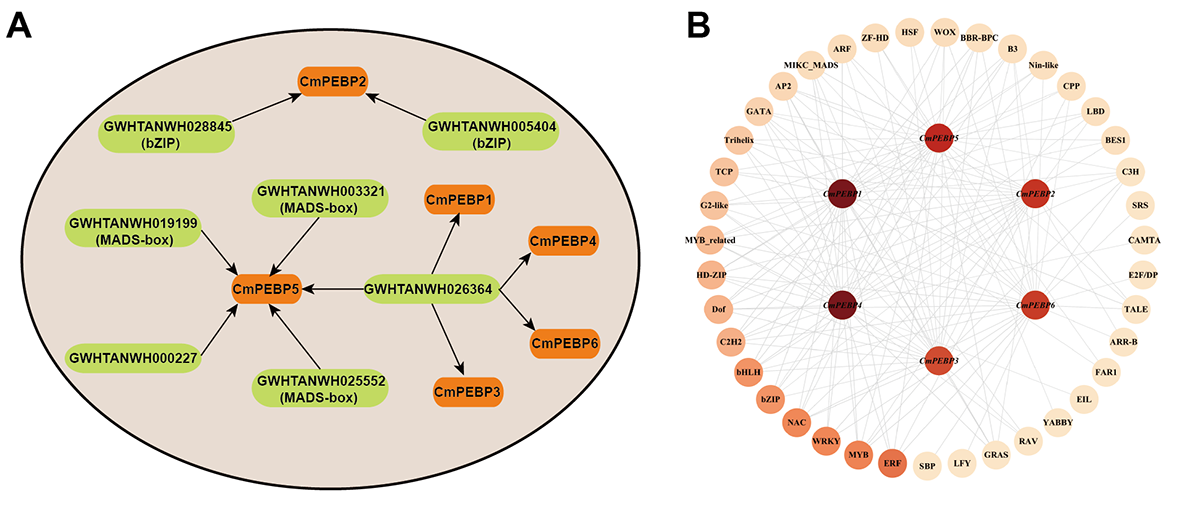

Supplement: Supplementary file 2 [file Image2.tif]

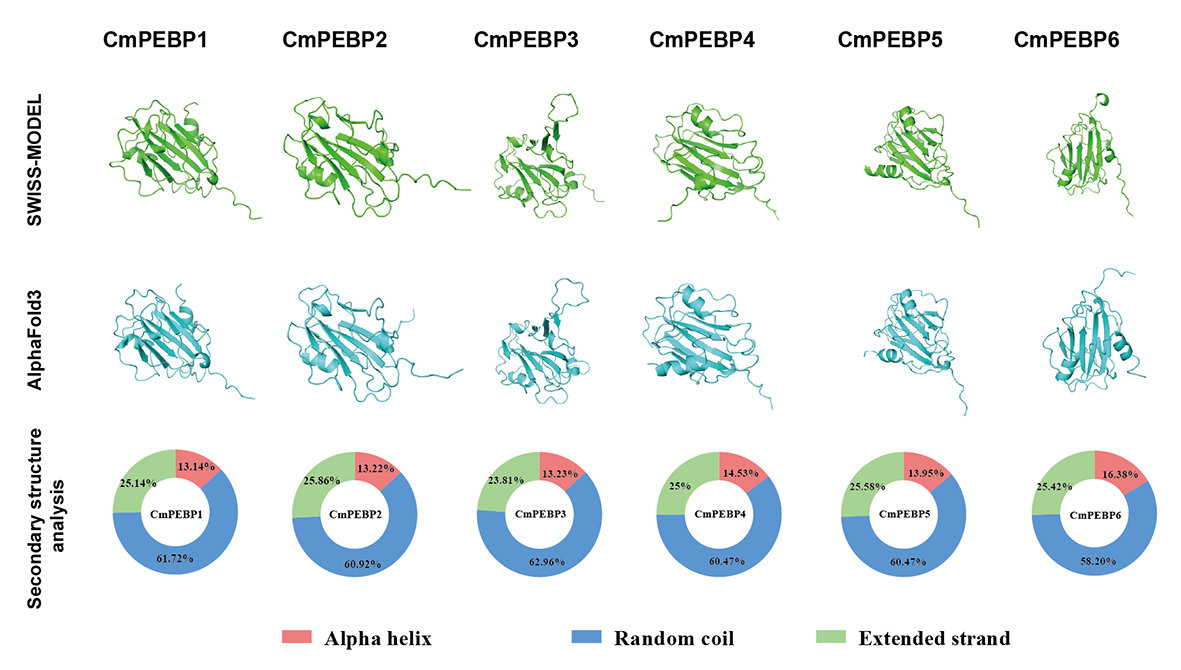

Supplement: Supplementary file 3 [file Image1.tif]
